# Mapping climate discourse to climate opinion: An approach for augmenting surveys with social media to enhance understandings of climate opinion in the United States

**DOI:** 10.1371/journal.pone.0245319

**Published:** 2021-01-14

**Authors:** Jackson Bennett, Benjamin Rachunok, Roger Flage, Roshanak Nateghi

**Affiliations:** 1 School of Industrial Engineering, Purdue University, West Lafayette, Indiana, United States of America; 2 Ecological Sciences and Engineering, Purdue University, West Lafayette, Indiana, United States of America; 3 Department of Safety, Economics, and Planning, University of Stavanger, Stavanger, Norway; STL UMR8163 CNRS, FRANCE

## Abstract

Surveys are commonly used to quantify public opinions of climate change and to inform sustainability policies. However, conducting large-scale population-based surveys is often a difficult task due to time and resource constraints. This paper outlines a machine learning framework—grounded in statistical learning theory and natural language processing—to augment climate change opinion surveys with social media data. The proposed framework maps social media discourse to climate opinion surveys, allowing for discerning the regionally distinct topics and themes that contribute to climate opinions. The analysis reveals significant regional variation in the emergent social media topics associated with climate opinions. Furthermore, significant correlation is identified between social media discourse and climate attitude. However, the dependencies between topic discussion and climate opinion are not always intuitive and often require augmenting the analysis with a topic’s most frequent n-grams and most representative tweets to effectively interpret the relationship. Finally, the paper concludes with a discussion of how these results can be used in the policy framing process to quickly and effectively understand constituents’ opinions on critical issues.

## Introduction

Enacting sustainable climate policy requires understanding public opinions on climate change. This is complicated by geographic variations in climate change belief in the United States, where county-level belief in climate change ranges from 43-80% of the population [1]. These variations have been attributed to endogenous factors such as regional heterogeneity in racial and ethnic composition [1], media consumption [2], and the beliefs of an immediate social circle [3]—as well as exogenous factors such as the frequency of exposure to natural disasters [4, 5] and geographic proximity to the coast [6]. Understanding causes of variation in climate change opinions requires a holistic analysis of the topics, themes, and events associated with climate change beliefs. In this paper, we focus on modeling the dominant topics, themes, and events present in social media discourse which correspond with regional, survey-based measures of climate change belief to augment surveys and provide additional depth to the opinions on climate change gathered by surveys.

Previous survey-based research provides a baseline understanding of climate change attitudes at a regional level [1, 7], however, it falls short of capturing the complexities of individual climate change belief. In response to survey questions, individuals must reconcile and ultimately map their beliefs regarding sustainability and climate change to a suitable survey response [8]. This process emphasizes an individual’s response to the question rather than the complexities which contribute to it [9–11]. Consequently, surveys provide a barometer for public opinion, but lack the detail necessary to explain individual motivation and activity [12].

Recent research has utilized social media activity to form deeper insights into individual motivations and perceptions in an effort to further understand the beliefs surrounding climate change [7, 13–15]. Social media is a platform for opinion sharing, and provides unique access to the complexities of an individual’s opinion [16]. On microbloging sites such as Twitter—with explicit content-length limits—all discussion is inherently distilled by the user, producing messages which are dense with relevant content [7]. The simplification of content combined with low barriers to entry has led to the use of social media as a tool for understanding the specifics of climate change belief [4, 7, 13, 14, 16–19]. However, social media analysis has been criticized for an overemphasis on content rather than understanding the constituent factors which contribute to an indivdual’s online content [13, 15].

In this paper, we propose a framework, grounded in statistical learning theory and natural language processing, to harness both survey and social media data in order to relate regional opinions on climate change to the dominant set of topics, themes, and events which may underlie them. We show that the social media-detected topics, themes, and events which color climate change opinions demonstrate regional heterogeneity in the continental United States. Using Latent Dirichlet Allocation (LDA), we distill the social media discourse into a set of topics constituting the landscape of climate change discussion. These regional portfolios are then mapped onto survey-based responses on climate change issues to explore the relationship between opinion and discourse. To our knowledge, this is the first rigorous integration of social media discourse with survey data in the context of climate change.

## Data and methods

This analysis seeks to answer the question: “How can social media activity be integrated with survey responses to improve the characterization of climate change opinion?” In other words, the goal of this study is to map Twitter activity onto survey responses to uncover the complex dependencies between discourse and opinion. To do this, we augment climate opinion survey data by developing a predictive model which utilizes a summarized portfolio of social media data as input to predict climate opinion. We then assess the partial dependencies between the input and output data to deduce the effect of individual topics on climate opinions. In this section, we describe both the social media and climate survey data used for this analysis, outline the structure of the predictive modeling framework, and describe the statistical validation performed on the data.

### Data

Two key sources of data were used in this study: climate change opinion survey data and social media activity, aggregated by US county. Climate opinion survey data were sourced from the Yale Climate Opinion Dataset [1] while social media data (i.e. tweets) were sourced from Twitter.

#### Climate opinion dataset

Survey research is a time-intensive and expensive method for gathering information on public opinion. To combat this challenge, in 2015 Howe et al. [1] developed a model to predict survey responses on a variety of climate related issues. The topics covered in the survey range from risk perceptions to policy preferences. The model provides information at the state, congressional district, metropolitan, and county levels, using a small set of demographic and geographic variables [1]. Based on validation on several independently conducted surveys, the model is reported to be accurate to within seven percentage points: slightly less accurate than the typical three percentage points expected of a traditionally-conducted survey [1]. While lower in accuracy, it provides the advantage of access to highly granular and spatially expansive data. For this analysis, the predicted survey responses are used as the response variable, as the granularity of the data allows for mapping the high resolution geo-located social media data to climate opinions. With this data source, climate opinion information can be mapped at the county rather than state level and ultimately provides more data for training and validation of the proposed predictive model.

#### Twitter data

To capture the topics, themes, and events associated with regional climate opinions, we extract data from Twitter user accounts. Twitter offers two well-documented Application Program Interfaces (APIs) through which data can be accessed. The Standard API allows virtually unrestricted access to tweets produced in the last seven days, with a limit on the number of requests per hour. The Premium API allows users to access the full Twitter archive, but has much more restrictive rate limits. This API is designed for users and organizations who are interested in a paid subscription.

In the interest of data accessibility, this analysis relies on the Standard API. By using tweets collected over a week long period, we ensure that all of the information required to conduct the study can be collected without a premium API subscription. One shortcoming of the Standard API is that it is not guaranteed to return every tweet in a given period of time. However, by taking advantage of different parameters in Twitter’s search function (namely specifying the ID of the most recent tweet to retrieve), the majority of tweets within a specific window can be retrieved. In two validation tests conducted over two separate three hour windows, we found that the Standard API returned at least 98% of the tweets retrieved by the Premium API.

We utilize two data subsets in training the proposed predictive model: a large corpus to train the topic model (later referred to as the topic corpus) and a smaller, geo-located corpus to derive regional topic features (referred to as the regional corpus). Because of Twitter’s privacy policy, not all tweets can be geo-located, which leads to the reduction in size from the topic to regional corpus. However, as tweets are generally short, it is important to maximize the sample size used to develop the topic model, hence the two distinct corpora.

The topic corpus consists of every tweet that matches the keywords “carbon” (excluding “carbon monoxide”), “climate”, or “global warming” (and excludes “RT”, Twitter’s representation of a retweet) in the region of interest (US, in this case) over a seven day period. In this study, the date range is April 18^th^ through April 25^th^, 2019—notably encompassing Earth Day and the Extinction Rebellion in London. The following is an example query to illustrate how data was collected, modified to enhance readability:

query = (global warming) or (climate) or (carbon)

excluding (monoxide) and (RT)

count = 100

tweet_mode = extended

max_id = [most recent tweet id]

where *query* is the specific keywords being targeted, *excluding* specifies words to exclude from the results, *count* identifies the number of tweets to return in the query (100 is the maximum), *tweet_mode* describes whether or not data beyond the tweet itself should be returned, and *max_id* specifies the most recent tweet ID to be used in the query (allowing us to exclude tweets that have already been collected, as tweet IDs are issued chronologically.

After data acquisition, the final topic corpus includes roughly 350,000 tweets. The regional corpus consists of geo-located tweets that match the aforementioned criteria. The most precise form of geo-tagging is a tweet that includes the latitude and longitude where the tweet originated, but less than 1% of tweets include this information. Another form of geo-tagging is a tweet that is associated with a specific location or a tweet that originates from a user associated with a specific location (e.g. Chicago, Illinois). For these tweets to be returned by the query, the entire region as specified by Twitter must be encompassed by the search radius. To perform geo-tagging, the search criteria is updated to include geographic coordinates and a search radius as follows:

geo-code = latitude, longitude, radius

where *latitude* and *longitude* specify the geographic center of a county and *radius* is the radius from the center in which to look for tweets. For the tweet to be returned by the query, it must be geo-tagged. The final regional corpus includes 190,000 tweets.

### Topic modeling

#### Latent Dirichlet Allocation (LDA)

To extract the key topics in the corpora of Twitter data, we utilize an unsupervised clustering technique called Latent Dirichlet Allocation (LDA). Latent Dirichlet Allocation (LDA) has been used extensively to detect topics on Twitter [20]. LDA is a probabalistic topic model, a class of Bayesian latent variable models. At a high level, it represents documents as a mixture of topics which are in turn represented by a set of words [21]. Given a corpus of documents, an LDA model learns the topic representation of each document and the words associated with each topic. Once a model is trained, given a document, the model will produce a topic likelihood distribution which identifies the relevant topic(s) in a document. For our analysis, we use a popular Natural Language Processing (NLP) Python package, Gensim [22] with the Machine Learning for Language Toolkit (MALLET) implementation [23].

We note there are a significant number of different methods for extracting topics from large text corpora, each with their own advantages and shortcomings. In the case of LDA, the algorithm assumes that each document contains multiple topics. However, in the case of tweets, which are likely to contain a limited number of topics due to character restrictions, this may not be a reasonable assumption. Previous research has proposed a modified LDA algorithm, namely Twitter-LDA, which assumes that each tweet contains exactly one topic [20]. In a comparison between LDA and Twitter-LDA on unfiltered Twitter data, where the number of topics was set at 110, the Twitter-LDA outperformed standard LDA [20]. However, the rationale for using standard LDA in this study is two-fold: **(1)** the goal of the LDA analysis is to derive topic distributions for distinct geographic regions; as this is an aggregate measure, by assuming that each tweet contained exactly one topic, we would limit the components of the topic distribution, potentially reducing the accuracy of the overall distribution, and **(2)** because the data is filtered to only pertain to climate change, the topics identified are likely to have more overlap than in a general tweet corpus and thus it was not reasonable to assume that each tweet would only contain a single topic. Here we utilize LDA as a topic model, but we emphasize that the approach presented in this study is not dependent on that specific method. In summary, the choice of the algorithm should be guided by the research question and available data.

#### Data preprocessing

Latent Dirichlet Allocation (LDA) requires preprocessing each of the incoming documents. We begin this process by removing punctuation, URLs, and mentions (i.e. users referencing other users) from tweets. Though this information can be important for certain applications with Twitter data, it offers little value in the development of a topic model. After removing these features, we convert the remaining text to lowercase. Next, we remove any stopwords from the document. Stopwords (e.g., “the”, “and”, “are”, etc.) are the most common words in the English language and have little bearing on the overall meaning of a document [24]. Additionally, we remove stopwords specific to Twitter discussion. Examples include ‘&amp;’ (the Twitter rendering of the ampersand sign) and ‘RT’ (Twitter code indicating that a tweet is a retweet, or copy, of another user’s tweet). We also remove the words “climate” and “carbon” as well as the phrase “global warming” as those were the keywords contained in the query used to construct the dataset and would otherwise be over-represented in every topic. Finally, we reduce each word to its lemma, or root. This ensures that the algorithm does not incorrectly identify different tenses or forms of a word as separate words. To perform these tasks, we use the the NLTK package in Python [25].

Following this preliminary preprocessing, we take further steps are to increase the efficiency at which the model is developed. Notably, we remove words that occur very frequently and those that occur infrequently from the set of words (i.e., dictionary) which is used to construct the topic model. Reducing the size of the dictionary, significantly improves the run-time of the algorithm, allowing for rapid testing and iteration. To reduce the size of the dictionary, we first only consider words greater than two characters. We then eliminate words that appeared in greater than 50% of tweets (e.g., “the”) as they would provide little information about the relevant topic. Next, we drop words that appeared in fewer than 100 (less than 0.03% of the dataset) of the tweets.

Finally, we leverage a randomized subset of the tweets used in preliminary model training for identification of the optimal number of topics. The size of the subset depends on the phase of model development and is specified in the following section.

#### Final topic model

Topic modeling requires user input for determining the optimal number of topics. In a well-performing model, the topics are distinct and intuitive. There are a variety of metrics that can assess different aspects of model quality but there are no formalized methods to make a holistic assessment. One popular metric is coherence, which rewards similarity within a topic and contrast between topics [26]. There are several ways to compute coherence and one of the most widely used metrics is *C*_*v*_ [26]. This metric combines several measurements, specifically, indirect cosine measure, boolean sliding window, and normalized pointwise mutual information, and has been shown to accurately indicate the degree to which a topic can be easily interpreted [26].

To determine the ideal number of topics, we iteratively construct models over a large range of topics with a step of two and computed coherence with the *C*_*v*_ metric. Based on these results, we select the range of topic numbers where coherence was highest and repeat the process with a smaller range and step size of 1.

Many topic models constructed with Twitter data have over one hundred topics in the final model [20]. However, as the tweets used in this analysis already have a prioi filtering related to climate change, we use lower ranges of topics as the training corpus is unlikely to contain as many topics as an unfiltered tweet stream. In the first round of model development, we test models with 10, 12, 14, …, 50 topics. Based on these results (depicted in S2 Fig in S1 File), we then test models with 14, 15, 16, …, 22 topics.

Based on the coherence score analysis, we identify seventeen as the appropriate number of topics in the model. This set largely encompasses the breadth of Twitter discussion on climate change while minimizing overlap between topics. The final topic model makes use of the entire 350,000 tweet corpus to ensure that the training data accurately represents the extent and variety of discourse on Twitter. A concise summary of the final topics is presented in Table 1.

**Table 1:** Categories from LDA topic analysis and a brief summary of their content.

Topic	Description
Climate Impacts	Adverse impacts, such as sea level rise, drought, forest fires, etc.
Earth Day	Celebration, activities, and tips related to Earth Day
Politicians	Political platforms of democratic candidates and the US President
Sustainability Promotion	Advice on reducing carbon footprint and greenhouse gas emissions
Activism	Climate activists (e.g. Greta Thunberg) and events (e.g. Extinction Rebellion)
Green New Deal	Commentaries on Congress’s Green New Deal proposal
Them, Not Us	How others are affected by and should respond to climate change
Clean Energy	Progress towards renewable energy targets and technologies
Environmental Justice	Concerns over environmental degradation, especially the impact of pipelines
Youth & Education	Role of parents and teachers in educating young people about climate change
Climate Denial	Arguments supporting the denial of climate change
Grassroots Action	Social media based efforts to solicit signatures for various petitions
Carbon Tax	Discussion of relationship between taxes and climate change
Weather Reports	Daily weather updates issued via Twitter
Global Warming	Arguments supporting global warming as a natural phenomenon
Trust in Science	Discussion of the importance of science in decision making
Plants, Trees	Relationship between plant life and climate change

It should be noted that the topic names and descriptions (Table 1) are subjective and represent our best effort at concisely describing the complexity of each topic category. A key challenge in LDA analysis is discerning the distinct characteristics of each topic set. The state-of-the art involves referring to the top n-grams and most representative tweets for each category (see S3 Table and S4 Fig in S1 File)—an inherently subjective task. While this analysis is based on quantitative representations of these topics, it is critical to acknowledge the subjectivity inherent in unsupervised topic models and its implications for the study’s inferences.

### Predictive model

We employ statistical learning methods to develop a predictive model relating Twitter discourse to climate opinion survey responses. The model takes the previously discussed twitter topic distributions as inputs to predict climate change opinions, as discussed in more detail below.

#### Response variable—first principal component, *CCO*

The Yale Climate Opinion Dataset [1] contains a variety of responses on various issues related to climate change, ranging from trust in scientists to opinions on renewable energy policies. Rather than developing separate models for each response, we leverage dimensionality reduction techniques and consolidate all of the survey responses into a single variable using Principal Component Analysis (S3 Fig in S1 File).

The first principal component accounts for 89% of the variance in the data, indicating that the survey responses are highly correlated. This is not surprising since the survey questions speak to people’s beliefs related to climate change; those that believe climate change is a pressing issue are also likely to believe that policy addressing these issues is important. Because of the extremely high correlation between variables, it is reasonable to consolidate them into a single index which we coin as “Consolidate Climate Opinion (*CCO*)”.

**Fig 1:**
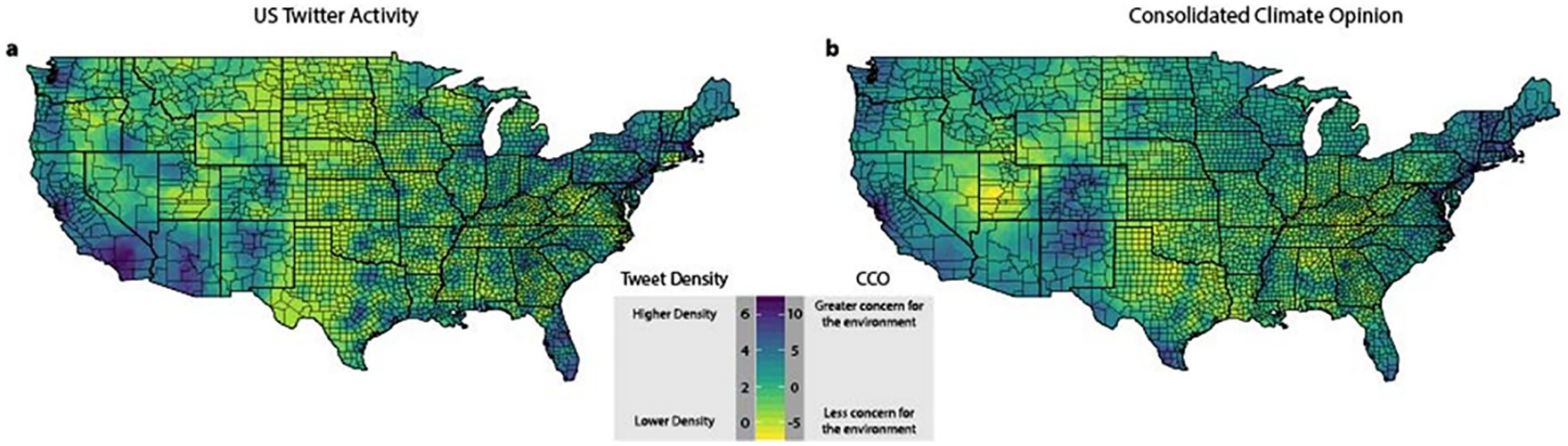
Distribution of Consolidated Climate Opinion (CCO) and Twitter users in the United States. (a) County-level CCO, calculated as the principal component reduction of 14 survey questions collected in the Yale Climate Opinion Dataset [1]. (b) County-level distribution of Tweet Density (tweets / person, log scale) in the United States based on climate-related tweets collected using the Twitter API from April 18^th^ through April 25^th^, 2019.

We posit that *CCO* represents the general degree of concern for the environment and climate change related issues. Fig 1a displays tweet frequency across the nation based on climate-relevant Twitter activity collected over this period. Fig 1b shows geographic variation in climate attitudes. Based on the data used from the Yale Climate Opinion Survey (S2 Table in S1 File), regions with higher *CCO* values have residents who are more worried about climate change and its impacts as well as more likely to support pro-environmental regulations and policies.

#### Predictors—topic distributions

To develop topic distributions for each county, we process tweets from the regional corpus using the topic model developed from the full corpus. For each county in the U.S., we filter the dataset for tweets associated with that county. We then preprocess each tweet in the filtered subset is then preprocessed in the manner described earlier. The output of an LDA topic model is a set of numbers which represent the probability that a given word from each tweet belongs in each topic. We then sum this set of numbers is then summed across all of the words to derive a so called “topic distribution” for the tweet.

To create the final county distribution, we sum topic distributions for all tweets in a county. We normalize each county distribution by dividing by its sum such that the sum of the final county distribution is one, allowing for comparison between counties. The analysis excludes counties with four or fewer tweets as the distributions generated from such a small number of tweets are not accurate representations of the county’s beliefs.

#### Model development & assessment

We use a random forest model [27] to map *CCO* on topic distributions. Random forest is a tree-based, non-parametric model. To make predictions, the random forest algorithm builds *b* decision trees and averages the estimates of all of the trees to create a final estimate of the response variable (*CCO*) as a function of the topic distribution observed in the county of interest. This is mathematically expressed as:

$$\begin{array}{c} \hat{f}\left(x\right)=\frac{1}{B}\sum_{b=1}^{B}T_{b}\left(x\right) \end{array}$$

where *T*_*b*_ is an individual regression tree and *x* represents the input data. The random forest algorithm is effective in modeling data that exhibits complex, nonlinear relationships due to its flexibility and lack of assumptions about the underlying structure of the data [27].

The focus of this analysis is characterize the regional differences in the relationships between tweet topic distribution and survey response. A significant amount of prior literature has demonstrated that beliefs in climate change vary significantly throughout the country [1, 28] and a natural extension of previous results is to develop models for each region of the continental US. If beliefs in climate change vary by region, it follows that the relationship between social media topic portfolios and *CCO* would also vary regionally. Furthermore, the development of regional models can help extract nuanced relationships that may otherwise be masked in a larger, national model. Specifically, we develop a model for the Northeast, Midwest, Southeast, Southwest, Pacific, and Rocky Mountain regions.

A key component of building each of the final six regional models is variable selection, as including too many variables in a model can lead to overfitting or inaccurate inferences on the relationships between predictors and response [29]. Here, we employed the Variable Selection Using Random Forests (VSURF) algorithm [30]. This is a data-driven algorithm that ranks the importance of every candidate variable in making a prediction. VSURF defines importance as the mean reduction in mean square error observed by including a variable in the model. A variable which leads to a greater reduction in error is ranked as more important. To determine the ranking, VSURF repeatedly builds models and averages the variable importance from each iteration. Beginning with the most important variables, models are built by step-wise addition of variables in order of decreasing importance. Once the improvement in model accuracybecomes negligible, the process ends and the most important variables are returned. We developed final models for all regions using the key variables identified by the VSURF algorithm.

In addition to reducing overfitting, variable selection also improves model interpretability [29]. Though random forest models are non-parametric, there are a wide variety of tools available to characterize the relationship between predictors and response. To characterize the relationship between predictors and response for each model, we use partial dependence plots. These plots show the effect a predictor variable on the response while the effects of the other variables in the model are accounted for [31]. Mathematically, the relationship of the predictor on the response is given as:

$$\begin{array}{c} \hat{f_{j}}\left(x_{j}\right)=\frac{1}{n}\sum_{i=1}^{n}\hat{f_{j}}\left(x_{j},x_{-j,i}\right) \end{array}$$ (1)

where $\hat{f_{j}}$ represents the trained model, n is the number of observations in the training set, and *x*_−*j*_ is all variables other than *x*_*j*_ in the training set. In order to evaluate the generalization performance of each regional model}, we conduct randomized partitioning of the data into training and test sets such that 80% of the data is used for training, and 20% for testing. The regional models are then built using the training set and validated by making predictions using the test set. Model performance is evaluated using normalized root mean square error. Model performance results are presented in the Table 2 below.

**Table 2:** Model performance based on Normalized Root Mean Square Error (NRMSE) and *R*^2^ for each regional model.

Region	In Sample			Out of Sample		
	NRMSE	*R*^2^	Impr. Over Null (%)	NRMSE	Correlation	Impr. Over Null (%)
Midwest	39.7	0.96	60.2	73.1	0.73	26.4
Southwest	42.6	0.96	57.1	72.1	0.77	26.5
Southeast	40.4	0.96	59.5	78.7	0.62	20.8
Northeast	35.0	0.96	64.9	61.1	0.80	38.2
Pacific	39.9	0.96	59.9	70.4	0.77	27.6
Rockies	44.2	0.95	55.5	73.1	0.73	24.8

Models with lower NRMSE and higher correlation have more predictive power. Additionally, we show the percent improvement over the mean-only, or null model. A higher percent improvement indicates increased predictive power.

These results show that the models perform better on in-sample data as compared to the out-of-sample data, which is to be expected as the models were trained on the in-sample data. In this study, we are primarily interested in in-sample performance as we are seeking to explain the available data rather than generalize to new use cases. However, including the out-of-sample performance is important as it demonstrates that the models don’t overfit the data and possess reasonable predictive power. Additionally, each model was compared to the null (mean-only) model as a benchmark for the performance of statistical regression models. The null model does not rely on leveraging a statistical model (based on supervised learning) to estimate the response variable as a function of the independent variables; instead it assumes that the best predictions can be achieved by simply calculating the historical average value of the response variable. Table 2 shows that in sample, the models outperform the null model by an average of 59.5% and outperform the null model by an average of 27.4%, indicating that they are successfully identifying relationships in the data.

## Results

### Regional topic portfolios

**Fig 2:**
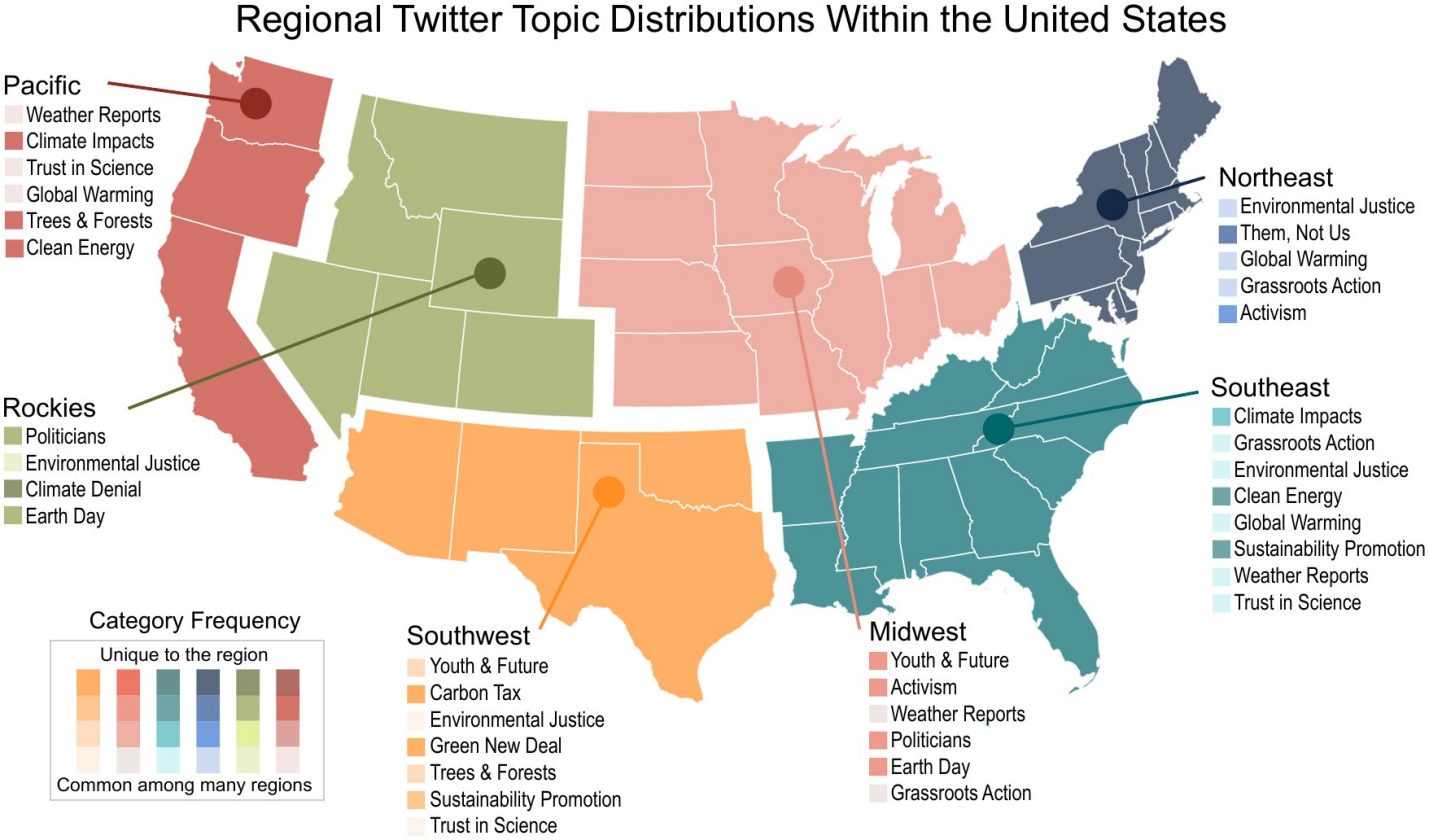
Regional Twitter topic distributions. Each region of the US used in this analysis is shown along with its corresponding most important LDA topics. Topics are listed in order of their importance (top to bottom), and colored based on how frequently they occur in regional discussion: darker is more unique to the region while lighter is more common nationally.

In order to investigate how the attributes associated with climate change opinions differ across regions of the US, we develop statistical models for each region of the US relating Twitter topics to Consolidated Climate Opinion (*CCO*). As previously described, we identify the subset of Twitter topics which best predict *CCO* and construct models for each region using only that subset. The topics included in the model and the regional breakdown are depicted in Fig 2, which illustrates the regional differences in topic portfolios used to predict *CCO*. In addition to reaffirming previous research that opinions on climate change vary regionally, this analysis highlights that the topics which are important to predicting *CCO* in one region are not necessarily important in predicting *CCO* in a different region.

Many of the topic portfolios reflect regionally distinct trends contributing to climate opinions. One such topic is *Climate Impacts* in the Pacific as compared to the Southeast. These regions have recently experienced a disproportionate number of natural disasters [32, 33] which has been previously shown to influence climate change beliefs of a region [4, 5]. Similarly, *Environmental Justice* emerges as an important topic in the Rockies, Southeast, Southwest, and Northeast. We hypothesize that the importance of this topic in these regions is driven by the concerns surrounding the Keystone and Atlantic pipelines raised by activists and locals who fear the projects will negatively impact the environmental quality of their communities.

Another result is the importance of the trustworthiness of climate science across all regions, either as *Climate Denial*, *Global Warming*, or *Trust in Science*. Though the framing and language of these topics is distinct, they all include tweets that question or promote trust in the scientific community.

Additionally, there are topics which are focused on a specific event or policy. Event-related topics include *Activism* which primarily discusses London’s Extinction Rally and Swedish activist Greta Thunberg, and *Earth Day* which largely focuses on activities and events that occurred on April 22^nd^, 2019 celebrating environmental protection. Policy-related topics include *Green New Deal* which largely focuses on the eponymous House bill, and *Carbon Tax*, a discussion centered on Canada’s recently proposed carbon tax legislation. Because these categories deal with ephemeral subjects, we do not believe they are as relevant to discussions of long term trends in the climate debate. We do include them in this study, however, to demonstrate the rapidity and flexibility of social media mining for developing a regional analysis of climate change opinions. Finally, the *Weather Reports* category, which is composed nearly exclusively of daily weather updates delivered via Twitter, can be used as a loose proxy for an area’s adoption of technology but is not particularly informative in framing climate policy.

Because relevant issues and topics are dynamic and change over time, an important consideration with this work is which topics in this model speaks to long-term concerns, and which are in response to specific events. As a means of sensitivity analysis, a separate Twitter corpus was collected from the 15^th^ to the 21^st^ of March, 2019, which precedes the corpus used in this analysis by almost exactly one month (S1 File). By comparing both time periods, we demonstrate that the only topics that differ between the two corpora are those which are specific to an individual event or policy—such as *Green New Deal*, *Carbon Tax*, and *Earth Day*.

### Motivating the importance of context

**Fig 3:**
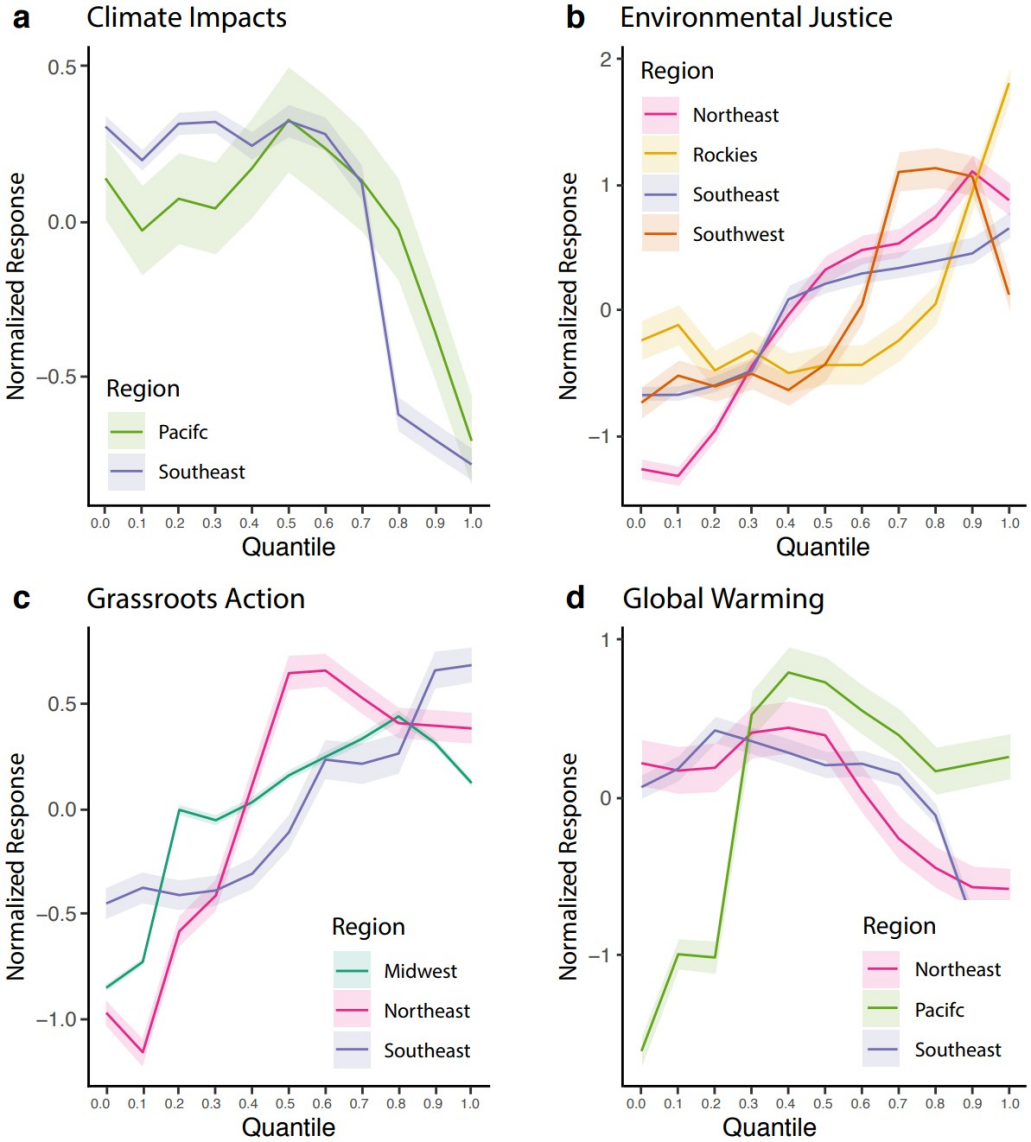
Partial dependence plots of topics and CCO. Each plot shows how CCO changes with an increase in a given Twitter topic while controlling for other variables. Shaded regions around line indicate a 97.5% confidence interval.

A key insight gained from the mapping of Twitter topics to *CCO* is how the topic portfolios associate with *CCO*. To understand the degree of contribution of each ‘key’ topic to *CCO*, we employ partial dependence plots [31], which isolate the effects of individual topics on climate opinions. Fig 3 shows the partial dependence plots for the top four of the seventeen topics included in the final model and their impact on climate opinions. Plots for the remaining topics considered in the model can be found in S1 Fig in S1 File.

The partial dependence plots reveal two key insights: (1) simply identifying the topics discussed in different regions is not sufficient to understand the reasons for which communities hold specific opinions on climate change, and (2) the relationship between topic portfolios and *CCO* varies between regions. Discussion of topics like Environmental Justice (**3b**) and Grassroots Action (**3c**) is generally positively correlated with *CCO*. However, the plot based on Climate Impacts (**3a** shows that the amount of discussion of the topic has little correlation with *CCO* below a certain threshold. Once this threshold is exceeded, a strong negative correlation emerges. Furthermore, the Global Warming plot (**3d**) reveals that the correlation between discussion of a particular topic and *CCO* can vary regionally. Based on these plots, it is evident that techniques such as partial dependence plots offer additional insights on the relationship between a particular region’s *CCO* and the topics discussed there.

## Discussion

While partial dependence plots provide key insight on the relationship between topic categories and climate opinion, they fall short of describing the contents of each topic. Though the partial dependence plot of Environmental Justice demonstrates that CCO increases with discussion of this topic increases, effectively interpreting these results requires that we understand exactly what the topic entails. To do this, we analyze the most frequent n-grams—sets of n words which occur in sequence—and most representative tweet for each category. Here, we identify the relative frequency of the top 30 keywords (i.e., onegrams), bigrams, and trigrams for each topic, the results of which are presented in S4 Fig in S1 File. These n-grams are accompanied by the most representative tweets from each of the topics used in each region’s final model, presented in S3 Table in S1 File. By augmenting our understanding of each topic with such information, we can more effectively interpret the partial dependence plots to understand the nuanced relationship between climate talk and climate opinion throughout the US.

Based on the topics used in the regional models (shown in Fig 2), science is pervasive in the national Twitter discussion. However, Twitter users in some regions promote climate science while other regions have a strong framing of climate denial. For example, in the Southeast, the most representative tweet in the *Global Warming* category is:

The fake news media keeps telling lies about global warming but look now it’s snowing in Minnesota. NO GLOBAL WARMING NO GLOBAL WARMING

This sharply contrasts with the Pacific, where the most representative tweet for the category is:

There are ‘scientists’ who are confident that the earth is less than 10,000 years old. And there are ‘scientists’ who are confident that man doesn’t contribute to global warming or that global warming isn’t happening

The tweet originating from the Southeast illustrates an emotionally charged message, denying the existence of global warming while the tweet originating from the Pacific addresses the idea that scientific claims should be evaluated holistically. These two tweets paint very different pictures of the *Global Warming* discussion in two different regions of the country and illustrate that even though tweets may be topically similar, it is important to contextualize them to understand the state of climate discussion in the respective region.

The context in which the term “Global Warming” is used in these tweets also helps to explain some of the results from the partial dependence plots. Previous research has found that communities which frame the issue as “global warming” rather than “climate change” show less progressive stances on sustainability and generally spend time debating semantics rather than policy levers [18]. Consequently, we would expect an increase in the *Global Warming* category to correspond with a decrease in *CCO*. This trend is observed in the Northeast and Southeast, but the Pacific demonstrates an increase in CCO corresponding with an increase in the *Global Warming* category. To explain the reversal of this trend, it is necessary to interpret the most representative tweets. In the Northeast and Southeast, the tweets which best represent this category are largely inflammatory. They frequently question the validity of global warming when faced with cooler local temperatures and focus on presenting observations which they believe disproves the phenomenon. In the Pacific, however, the representative tweets are less confrontational and focus on peer to peer education. Tweets from this region of the country which fall into the *Global Warming* topic attempt to resolve the disconnect between individual experience and scientific observation with the ultimate goal of fostering the development of a more progressive climate attitude.

Both *Environmental Justice* and *Grassroots Action* have an intuitive relationship with *CCO* in that greater discussion of these topics in a region is associated with higher overall *CCO* (Fig 3b and 3c). We hypothesize the positive relationship stems from correlation between individuals who actively monitor the local environment, protest threats to its integrity and individuals likely to respond positively to national surveys of climate opinions. This positive relationship between attributes of individual belief and twitter topic prevalence also occurs in individuals who feel personally responsible for driving change in their local community, which is the unifying theme of the *Grassroots Action* discussion. This topic largely focuses on people who promote collective action by soliciting signatures for electronic petitions focused on climate action, and accordingly has a positive relationship with *CCO*.

We find other counter-intuitive dependencies between topic and CCO. Increases in the *Climate Impacts* category are associated with *lower* overall *CCO*. We initially hypothesized the opposite to be true as communities who are aware of and discuss the adverse impacts of climate change are expected to take a progressive stance on the issue because they are directly experiencing its consequences. However, deeper analysis of the most common bigrams (pair of words which occur together) and most representative tweets from the *Climate Impacts* category reveal that much of the discussion revolves around whether or not the currently observed impacts—sea level rise, drought, hurricanes, etc.—are a result of anthropogenic climate change or of a natural cycle. In this context, the inverse relationship between *Climate Impacts* and *CCO* is more intuitive. Higher levels of debate surrounding this topic indicate increased levels of uncertainty in the impacts of climate change, which is generally associated with communities that hold a less progressive climate opinions. Regardless of the topic in question, a notable observation is that gaining insight into these regional results requires interpreting partial dependence plots, the topics that are present, and representative tweets.

## Conclusion

In this study, we present a framework to augment survey data with social media data and apply it to the topic of climate change. Specifically, we use natural language processing to create county-level topic portfolios based on social media data. This is then used to develop regional predictive models which explore the relationship between Twitter topic discussion and climate change survey responses. This mapping between survey data and social media discourse allows researchers to harness social media data to contextualize survey responses and better understand why people hold the opinions they do.

The key finding from this study is that while the relationship between climate talk and climate opinion can be intuitive, it often requires a deeper understanding of the context that underlies the debate. There are many potential applications of these results and we believe one of the most promising uses of the insights generated by augmenting surveys with social media discourse is in the policy framing process. For example, should sustainability-motivated policies such as carbon taxes be introduced at a national level, decision makers in the Pacific can leverage the regional attribute portfolio by presenting the tax as a program that would incentivize the development of clean energy. Policy makers in the Northeast, on the other hand, might choose to present the proposal as a method promoting environmental justice by ensuring that individuals and organizations are held accountable for the long-term impacts of fossil-based energy use. Framing policies in a way that aligns the pressing issues with the beliefs, attitudes and values of the constituents can accelerate the speed at which climate policies are enacted and can ultimately improve the nation’s ability to adapt to and mitigate the impacts of climate change.

In this analysis, we demonstrate that regions with similar climate opinions can have distinct portfolios of social media topics, themes, and events. We augment climate change survey data with Twitter activity to elucidate the connection between survey responses and specific climate change topics. While this analysis pertains specifically to the climate change debate, we use a generalizable framework which can be extended to a variety of other application areas. In future work, we aim to demonstrate the ability of this framework to perform longitudinal assessments of climate opinion. Given a sufficiently large training sample conducive to identifying long-term trends in Twitter discussion, longitudinal trends could be monitored to understand changes in climate opinion. Another future avenue for this work is integrating survey design with social media analysis. Survey questions which elicit responses in line with the types of analysis performed on social media (e.g. asking individuals to describe themes and topics when using LDA) could strengthen the future integration of social media and survey analyses. By using methods based on large, publicly available datasets, we can begin to understand the relationship between social media discourse and public opinion to produce significantly more insight than is provided by surveys alone.

## Supporting information

S1 File (ZIP)

## References

[1] Howe PD, Mildenberger M, Marlon JR, Leiserowitz A. Geographic Variation in Opinions on Climate Change at State and Local Scales in the USA. Nature Climate Change. 2015;5(6):596–603. 10.1038/nclimate2583

[2] Nisbet MC. Communicating Climate Change: Why Frames Matter for Public Engagement. Environment: Science and Policy for Sustainable Development. 2009;51(2):12–23. 10.3200/ENVT.51.2.12-23

[3] Pearce W, Holmberg K, Hellsten I, Nerlich B. Climate Change on Twitter: Topics, Communities and Conversations about the 2013 IPCC Working Group 1 Report. PLOS ONE. 2014;9(4):e94785 10.1371/journal.pone.0094785 24718388 PMC3981832

[4] Cody EM, Reagan AJ, Mitchell L, Dodds PS, Danforth CM. Climate Change Sentiment on Twitter: An Unsolicited Public Opinion Poll. 2015;10(8):e0136092 10.1371/journal.pone.0136092 PMC4546368 26291877

[5] Roxburgh N, Guan D, Shin KJ, Rand W, Managi S, Lovelace R, et al Characterising Climate Change Discourse on Social Media during Extreme Weather Events. Global Environmental Change. 2019;54:50–60. 10.1016/j.gloenvcha.2018.11.004

[6] Milfont TL, Evans L, Sibley CG, Ries J, Cunningham A. Proximity to Coast Is Linked to Climate Change Belief. PLOS ONE. 2014;9(7):e103180 10.1371/journal.pone.0103180 25047568 PMC4105574

[7] Kirilenko AP, Stepchenkova SO. Public Microblogging on Climate Change: One Year of Twitter Worldwide. Global Environmental Change. 2014;26:171–182. 10.1016/j.gloenvcha.2014.02.008

[8] Pasek J, Krosnick JA. Optimizing survey questionnaire design in political science: Insights from psychology Oxford handbook of American elections and political behavior. 2010; p. 27–50.

[9] Cannell CF, Miller PV, Oksenberg L. Research on interviewing techniques. Sociological methodology. 1981;12:389–437. 10.2307/270748

[10] Krosnick JA. Response strategies for coping with the cognitive demands of attitude measures in surveys. Applied cognitive psychology. 1991;5(3):213–236. 10.1002/acp.2350050305

[11] Turner C, Martin E. Surveying subjective phenomena. vol. 2 Russell Sage Foundation; 1985.

[12] Glynn CJ, Herbst S, Lindeman M, O’Keefe GJ, Shapiro RY. Public Opinion. Routledge; 2015 Available from: http://ebookcentral.proquest.com/lib/purdue/detail.action?docID=1181617.

[13] Fownes JR, Yu C, Margolin DB. Twitter and Climate Change. Sociology Compass. 2018;12(6):e12587 10.1111/soc4.12587

[14] Kirilenko AP, Molodtsova T, Stepchenkova SO. People as Sensors: Mass Media and Local Temperature Influence Climate Change Discussion on Twitter. Global Environmental Change. 2015;30:92–100. 10.1016/j.gloenvcha.2014.11.003

[15] Palen L, Anderson KM. Crisis Informatics—New Data for Extraordinary Times. Science. 2016;353(6296):224–225. 10.1126/science.aag2579 27418492

[16] Auer MR, Zhang Y, Lee P. The Potential of Microblogs for the Study of Public Perceptions of Climate Change. Wiley Interdisciplinary Reviews: Climate Change. 2014;5(3):291–296. 10.1002/wcc.273

[17] Williams HTP, McMurray JR, Kurz T, Hugo Lambert F. Network Analysis Reveals Open Forums and Echo Chambers in Social Media Discussions of Climate Change. Global Environmental Change. 2015;32:126–138. 10.1016/j.gloenvcha.2015.03.006

[18] Jang SM, Hart PS. Polarized Frames on “Climate Change” and “Global Warming” across Countries and States: Evidence from Twitter Big Data. Global Environmental Change. 2015;32:11–17. 10.1016/j.gloenvcha.2015.02.010

[19] An X, Ganguly AR, Fang Y, Scyphers SB, Hunter AM, Dy JG. Tracking climate change opinions from twitter data. In: Workshop on Data Science for Social Good; 2014.

[20] Zhao, Wayne Xin and Jiang, Jing and Weng, Jianshu and He, Jing and Lim, Ee-Peng and Yan, et al. Xiaoming Comparing Twitter and Traditional Media Using Topic Models Advances in Information Retrieval, 2011

[21] Blei DM, Ng AY, Jordan MI. Latent Dirichlet Allocation. Journal of Machine Learning Research. 2003;3(Jan):993–1022.

[22] Řehůřek R, Sojka P. Software Framework for Topic Modelling with Large Corpora. In: Proceedings of the LREC 2010 Workshop on New Challenges for NLP Frameworks. Valletta, Malta: ELRA; 2010. p. 45–50.

[23] McCallum AK. MALLET: A Machine Learning for Language Toolkit; 2002.

[24] Silva C, Ribeiro B. The Importance of Stop Word Removal on Recall Values in Text Categorization. In: Proceedings of the International Joint Conference on Neural Networks, 2003. vol. 3; 2003. p. 1661–1666 vol.3.

[25] Bird Steven, Loper Edward and Klein Ewan. Natural Language Processing with Python. O’Reilly Media Inc (2009).

[26] Röder M, Both A, Hinneburg A. Exploring the Space of Topic Coherence Measures. In: Proceedings of the Eighth ACM International Conference on Web Search and Data Mining—WSDM’15. ACM Press; 2015. p. 399–408. Available from: http://dl.acm.org/citation.cfm?doid=2684822.2685324.

[27] Breiman L. Random Forests. Machine Learning. 2001;45(1):5–32. 10.1023/A:1010933404324

[28] Hamilton LC, Keim BD. Regional variation in perceptions about climate change. International Journal of Climatology: A Journal of the Royal Meteorological Society. 2009;29(15):2348–2352. 10.1002/joc.1930

[29] Guyon Isabelle and Elisseeff André An introduction to variable and feature selection Journal of machine learning research. 2003;3;1157–1182

[30] Genuer R, Poggi JM, Tuleau-Malot C. Variable selection using Random Forests. Pattern Recognition Letters. 2010;31(14):2225–2236. 10.1016/j.patrec.2010.03.014

[31] Friedman JH. Greedy Function Approximation: A Gradient Boosting Machine. The Annals of Statistics. 2001;29(5):1189–1232.

[32] Mann ME, Gleick PH. Climate change and California drought in the 21st century. Proceedings of the National Academy of Sciences. 2015;112(13):3858–3859. 10.1073/pnas.1503667112 25829537 PMC4386383

[33] Herring SC, Hoell A, Hoerling MP, Kossin JP, Schreck CJ III, Stott PA. Explaining extreme events of 2015 from a climate perspective. Bulletin of the American Meteorological Society. 2016;97(12):S1–S145.

