# Supplementary material for: Mapping climate discourse to climate opinion: An approach for augmenting surveys with social media to enhance understandings of climate opinion in the United States
Source: PLoS One. 2021 Jan 14;16(1):e0245319. doi: 10.1371/journal.pone.0245319 (PMC7808624; doi:10.1371/journal.pone.0245319)
Supplement: S1 File — (ZIP) [file pone.0245319.s001.zip › climate_survey_supporting_information_untracked.pdf]

S1 File — Mapping climate discourse to climate opinion: An approach for augmenting surveys with social media to enhance understandings of climate opinion in the United States

Sensitivity Analysis

To validate the topics identified in the corpus used in this analysis, a separate Twitter corpus was collected from March 15th to March 21st, 2019, which precedes the corpus used in this analysis by approximately one month. To identify the topics present in this "sensitivity corpus", the same procedure as outlined in the previous sections was used. Based on coherence score, eighteen was selected as the optimal number of topics; compared to seventeen in the primary corpus. Though topic content was never identical between the two corpora, the majority of topics mapped well onto each other. S1 Table summarizes the topics observed in the two corpora, as well as their union.

S1 **Table.** List of topics based on inclusion in analysis and sensitivity corpora

Analysis Corpus	Both	Sensitivity Corpus
Weather Reports	Global Warming	National Emergency
Green New Deal	Climate Impacts	Youth Strike – News
Carbon Tax	Them, Not Us	Youth Strike – Response
Earth Day	Politics	Spanish
	Youth and Future	Youth Strike – Impact
	Sustainability Promotion	
	Environmental Justice	
	Energy	
	Climate Denial	
	Nature and Agriculture	
	Activism	
	Science	
	Grassroots Action	

As previously mentioned, the topic categories did not perfectly map onto one another. For instance, the *Energy* category in the model corpus focused more on renewable energy than the sensitivity corpus, which also includes mentions of fossil fuels. The *Environmental Justice* category was more limited to drilling specifically in the sensitivity corpus, while the model corpus also focused on air and water quality. Despite these discrepancies, it is clear that over the course of a month, the discussion around climate change remained relatively constant. The notable exception to this is Twitter activity driven by specific events and policies. Specifically, the youth climate strike (which occurred on March 15th, 2019) and mentions of the U.S. national emergency only appear in the sensitivity corpus. Similarly, *Earth Day*, the *Green New Deal*, and the proposed *Canadian Carbon Tax* only appear in the model corpus. The sensitivity corpus also includes a Spanish category, which was filtered in the model corpus. The only other discrepancy is the *Weather Reports* category, which occurs only in the model corpus. Based on preliminary investigation, this topic was combined with the climate impacts category in the sensitivity corpus.

From these results we see that topics which are thematically driven — rather than event-driven — are stable over time. We hypothesize that the discussion of specific events would spike as the event occurs and eventually vanish, while issues that continue to be relevant would be be relatively constant points of discussion, albeit in slightly different forms. With this observation in mind, the majority of the discussion presented focuses on topics which appear in both corpora as they are believed to be more relevant to long term trends. To further validate the selected set of topics, future analysis should perform topic analysis on a tweet corpus with a larger temporal separation, such as six months or even a year.

S2 **Table.** Data collected in Yale Climate Opinion Survey used in this analysis

Variable Name
Estimated percentage who somewhat/strongly support setting strict limits on existing coal-fire power plants
Estimated percentage who somewhat/strongly support regulating CO2 as a pollutant
Estimated percentage who somewhat/strongly support requiring utilities to produce 20% electricity from renewable sources
Estimated percentage who somewhat/strongly support funding research into renewable energy sources
Estimated percentage who think that global warming is happening
Estimated percentage who think that global warming is caused mostly by human activities
Estimated percentage who believe that most scientists think global warming is happening
Estimated percentage who are somewhat/very worried about global warming
Estimated percentage who think global warming will harm them personally a moderate amount/a great deal
Estimated percentage who think global warming will harm people in the US a moderate amount/a great deal
Estimated percentage who think global warming will harm people in developing countries a moderate amount/a great deal
Estimated percentage who think global warming will harm future generations a moderate amount/a great deal
Estimated percentage who think global warming will start to harm people in the United now/within 10 years
Estimated percentage who discuss global warming occasionally or often with friends and family
Estimated percentage who hear about global warming in the media at least weekly
Estimated percentage who somewhat or strongly trust climate scientists as a source of information about global warming
Estimated percentage who think global warming will harm plants and animal species a moderate amount/a great deal

S3 **Table.** Most representative tweets for each of the categories used in each region's final topic model

S3-1 **Table.** Pacific and Rockies Regions

Region	Category	Tweet
Pacific	Weather Reports	SEATTLE WA WFO Apr 23 Climate: Hi: 60 Lo: 48 Precip: Trace Snow: Missing
	Climate Impacts	should be dangerously high, but they haven't increased much since 2000. If we plug current temperatures into his model it shows that we would have had to cap the carbon-dioxide emissions that cause the enhanced greenhouse effect about 18 years ago.
	Trust in Science	Great thread! Discussing a very important issue within the scientific community "Parachute science." Also touches on others, as bias when citing papers, who is really impacted by climate change & how do we benefit as scientists doing these studies. Sources also cited Thank you!!!!
	Global Warming	There are "scientists" who are confident that the earth is less than 10,000 years old. And there are "scientists" who are confident that man doesn't contribute to global warming or that global warming isn't happening.
	Trees & Forest	Trees are vital. As the biggest plants on the planet, they give us oxygen, store carbon, stabilize the soil and give life to the world's wildlife. They also provide us with the materials for tools and shelter.
	Clean Energy	Similar to other renewable energy sources, solar power use reduces carbon-emission significantly, which helps reverse climate change. It's called clean energy because the installation, and operation of solar components results in significantly fewer pollutants in the air
Rockies	Politicians	What I want to hear about from prospective candidates are actual, doable policies. Hate-filled rhetoric & propaganda are ridiculous & do nothing to educate the populace on how we will fix infrastructure, health care, education, climate change, gerrymandering, etc. Discuss policy.
	Environmental Justice	The AtlanticCoast fracked gas pipeline would cross three states, threatening climate, water, & communities. @BankofAmerica's annual meeting is this week – tell it to say NoACP & stop financing this environmental & human rights disaster!
	Climate Denial	Do you actually answer questions or is FO the extent of your input. Do you think evolution and climate science are understood and true.
	Earth Day	Happy Earth Day! Let's take care of our planet, and it will take care of us.

S3-2 **Table.** Southwest and Midwest Regions

Region	Category	Tweet
Southwest	Youth & Future	Wow for a teacher you're really uneducated & certainly unqualified to teach,no wonder our kids rank so low... believe in Climate Change hoax just to mention a few of ur lameness
	Carbon Tax	Considering it costs about a thousand dollars for the Government to "give" you back your own money. Who can possibly think this stupid stuff up? Meanwhile, carbon consumption per capita continues to rise!
	Environmental Justice	The AtlanticCoast pipeline is a reckless, climate-killing boondoggle that would cross 600 miles of WV, VA, & NC & threaten water & communities. Tell @BankofAmerica: Say NoACP & stop financing this environmental & human rights disaster.
	Trees & Forests	Typical passenger vehicle emits 4.6 tons of carbon dioxide/year... A single hardwood tree can absorb about 1 ton of carbon dioxide over *40 years*
	Sustainability Promotion	its so easy to reduce your carbon footprint. There are reusable grocery bags and produce bags. Coffee cups, water cups, UTENSILS ! STRAWS ! All for less than $10 and you keep SO MUCH plastic out of the landfills.
	Trust in Science	I trust many Geologists, I don't trust any of these "Climate Scientists" that use fake numbers to generate funding. Read up on Tim Patterson or Don Easterbrook. These are actual Scientists, not people like Michael Mann or Hansen.
Midwest	Youth & Future	More than 8 in 10 teachers and parents support teaching kids about climatechange. But in reality, it's not always happening: Fewer than half of K-12 teachers told us that they talk about climate change with their children or students.
	Activism	Greta Thunberg to address Extinction Rebellion protesters in London as number of climate activists arrested rises to 830
	Weather Reports	BROKEN BOW NE Apr 24 Climate: Hi: 79 Lo: 34 Precip: Trace Snow: Missing
	Politicians	There's a new generation that will be voting for a president the first time in 2020. I don't see them voting for someone like trump. Their voice & votes can make a major difference to vote a candidate in office who cares about climate change healthcare, social security & medicare
	Earth Day	We treat planet Earth ruthlessly unkindly and unethically. The resources we extract from this planet isn't our property but of the future generations. Stop killing Earth with unsustainable activities.
	Grassroots Action	I've signed the petition demanding urgent action to stop the climatecrisis now? Will you add your name?

S3-3 **Table.** Northeast and Southeast Regions

Region	Category	Tweet
Northeast	Environmental Justice	Access to clean water is a crucial human right that must be protected. We applaud the UN Human Rights Committee for demanding the Trump administration address the Flint water crisis amid the broader issue of climate justice for affected communities.
	Them, Not Us	It makes them poorer by taxing the hell out of working people to pay for dead beat freeloaders .Wealthy politicians and people get rich off the scam and the middle class pays for it all .Global warming is a man made hoax
	Global Warming	No thanks you liberal lunatic this will only create higher energy prices... global warming is not real it's the natural cycle of our planet just ask Al Gore after he created (hahahaha) the internet he created the global warming hoax..
	Grassroots Action	Every little action counts so do sign. Time is running out climatechange. Our leaders have let us down - people worldwide are taking action. Join me & @friends_earth to demand UK Government takes urgent climateaction.
	Activism	Protesters from the environmental group Extinction Rebellion glued themselves to the London Stock Exchange building on Thursday on the final day of protests that have caused mass disruption in the British capital.
Southeast	Climate Impacts	Climate change can be affected be man, but reality is climate has changed for over 4.5B years. The Sahara Desert was a jungle..look at it now.
	Grassroots Action	This woman was arrested at our Stockholm action on Monday. And today she's come to Malmö to support our action outside public service broadcaster @svtnyheter. We demand that the media tells the truth about the climate and ecological emergency
	Environmental Justice	A big part of the @CoryBooker campaign is that environmental justice goes beyond just climate change. But goes into the quality and protection of the air that we breathe, water we drink. A problem vulnerable communities like Flint and Denmark, know all too well.
	Green New Deal	It's time for nations to unite around an International Green New Deal
	Clean Energy	From a climate perspective, this is very bad news. What we need is increased investments in renewable energy and energy efficiency, not increased investments in fossil fuel infrastructure
	Global Warming	The fake news media keeps telling lies about global warming but look now it's snowing in Minnesota. NO GLOBAL WARMING NO GLOBAL WARMING
	Sustainability Promotion	When we come together, the impact can be monumental. On Earth Day, try to go green by making small changes that make a big difference. Make more sustainable choices, reduce your carbon footprint, conserve energy – work in your community toward making an environmental difference.
	Weather Reports	SHELBY COUNTY AIRPORT Apr 20 Climate: Hi: 62 Lo: 44 Precip: 0.07 Snow: Missing
	Trust in Science	Excellent analysis."Every month, climate scientists make new discoveries that advance our understanding of climatechange's causes & impacts.Research gives a clearer picture of the threats we already face and explores what's to come if we don't reduce emissions at a quicker pace

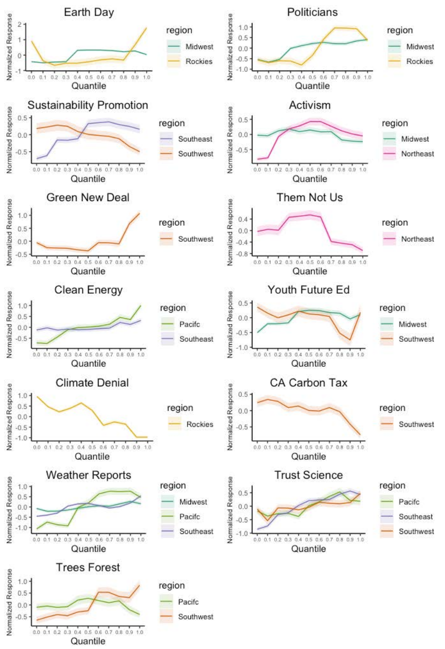

S1 **Fig.** Partial dependence plots for all topics not shown in main text. Quantile indicates how much the topic is discussed while Normalized Response shows the impact on CAI. Shaded regions indicate the 97.5% confidence interval.

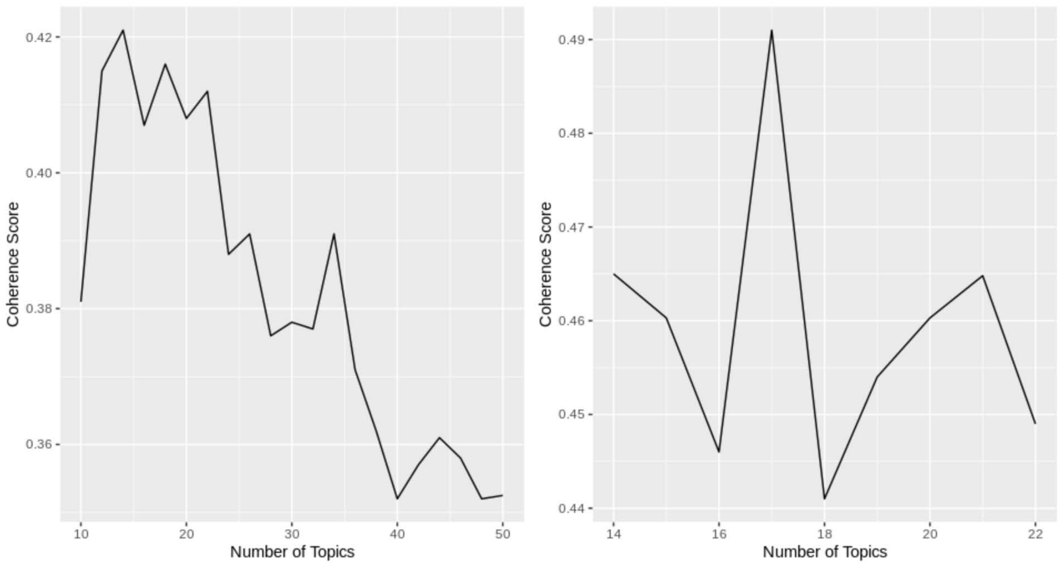

S2 **Fig.** Coherence scores used to determine the most suitable number of topics. On the left, results from models with 10-50 topics are shown. Based on these results, it is clear that the best performing region is 15-25 topics. The plot on the right shows results from models built in this range, from which it is clear that 17 topics produces the highest coherence score.

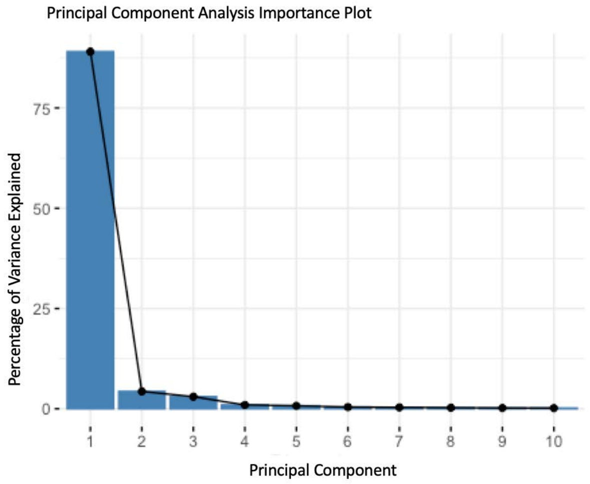

S3 **Fig.** Plot quantifying the variance explained by each principal component identified from Principal Component Analysis (PCA). The first principal component dwarfs all other principal components by more than order of magnitude, indicating that the variables included in the PCA are highly correlated.

S4 **Fig.** Top 30 onegrams (single words), bigrams (group of two sequential words), and trigrams (group of three sequential words) for each topic. Frequency measures the proportion of tweets within each topic that contain the n-gram (e.g. the bigram *extreme_weather* appears in 2.5% of the tweets in the *Climate Impacts* category. Note that due to quoting, some categories contain tweets with the same quoted text (notably *Politicians*), thereby augmenting the number of certain bi- and trigrams.

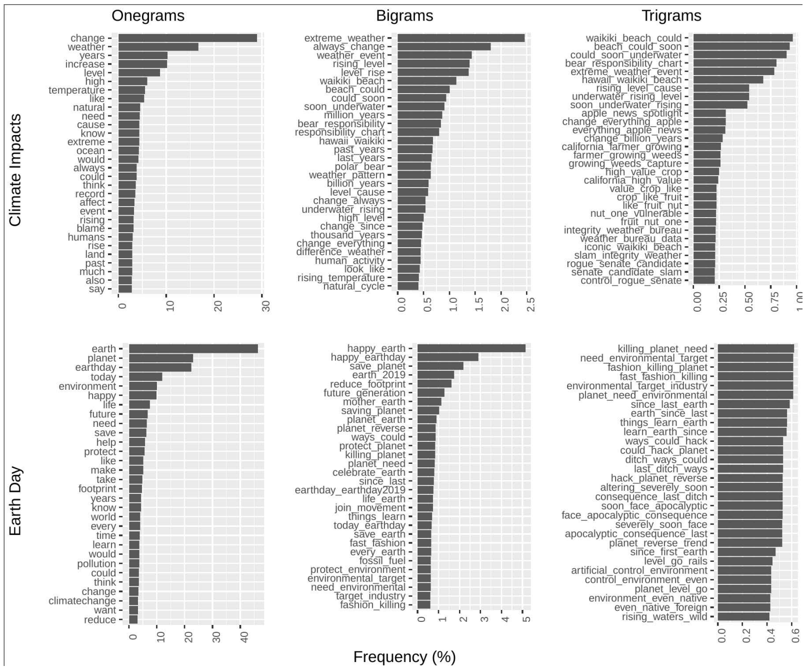

S4-1 **Fig.** *Climate Impacts* and *Earth Day*

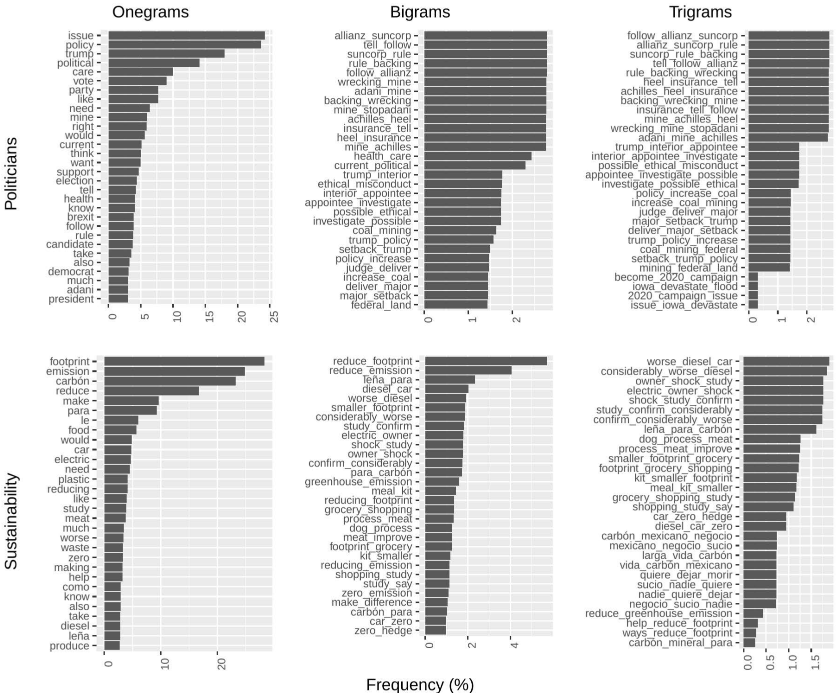

S4-2 **Fig.** *Politicians* and *Sustainability*

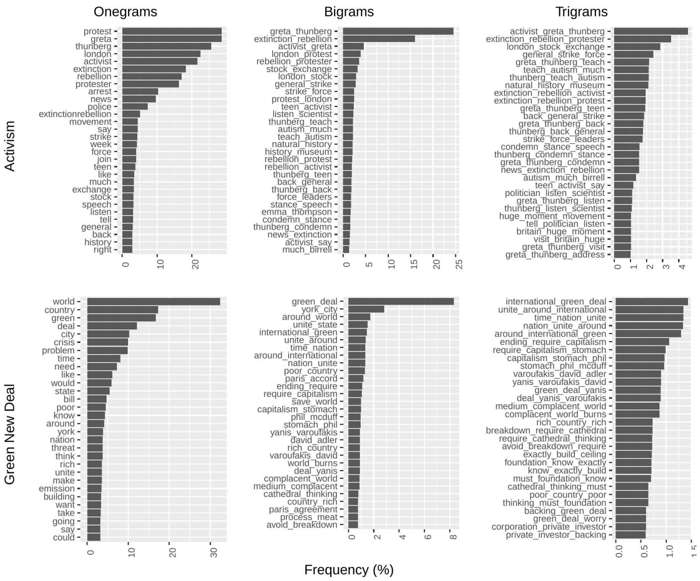

S4-3 **Fig.** *Activism* and *Green New Deal*

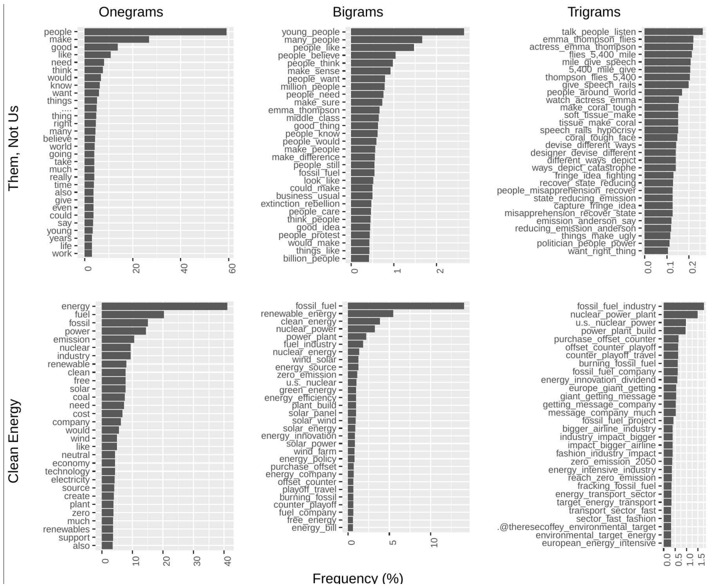

S4-4 **Fig.** *Them, Not Us* and *Clean Energy*

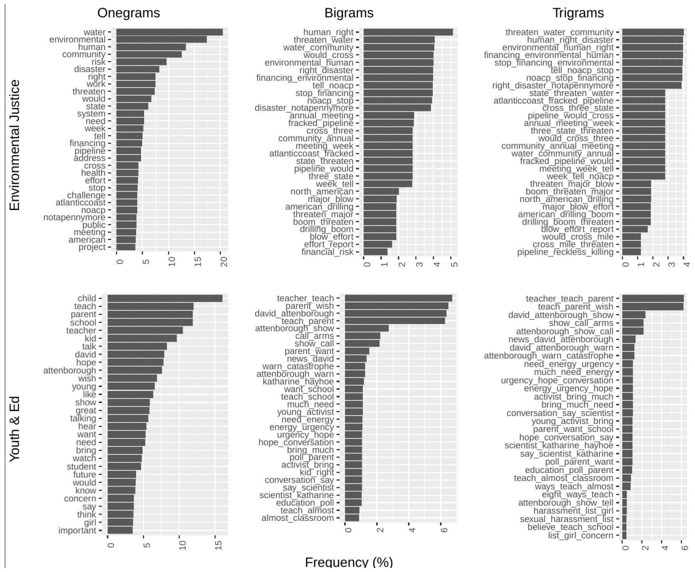

S4-5 **Fig.** *Environmental Justice* and *Youth & Ed*

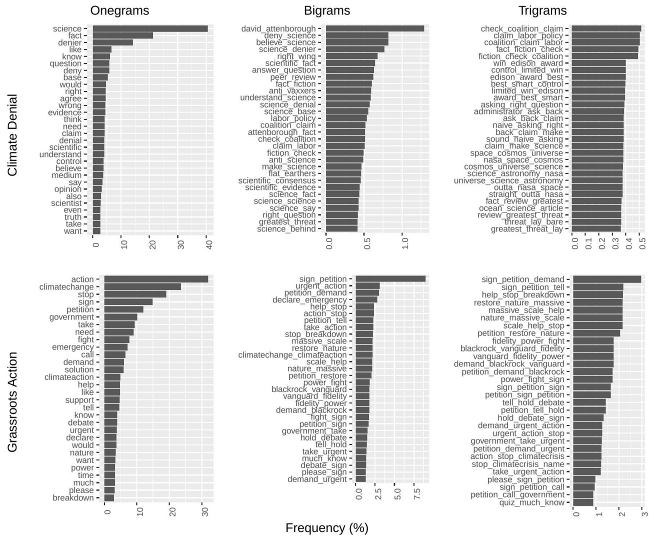

S4-6 **Fig.** *Climate Denial* and *Grassroots Action*

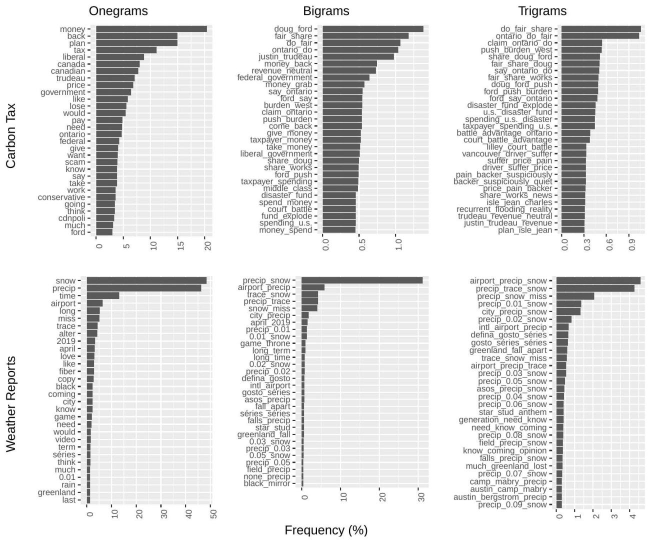

S4-7 **Fig.** *Carbon Tax* and *Weather Reports*

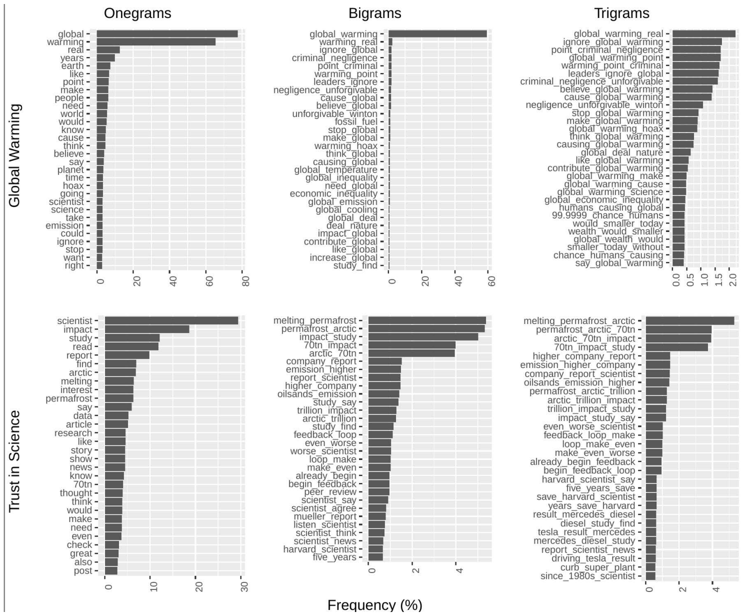

S4-8 **Fig.** *Global Warming* and *Trust in Science*

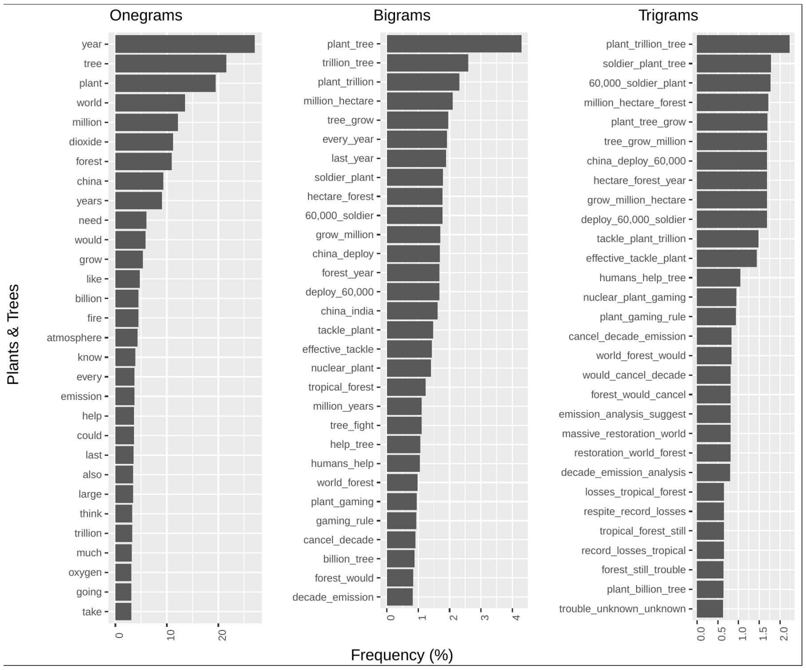

S4-9 **Fig.** *Plants & Trees*
